# Effect of Dentin Disinfection with 2% Chlorhexidine Gluconate and 0.3% Iodine on Dentin Bond Strength: An *in vitro* Study

**DOI:** 10.5005/jp-journals-10005-1440

**Published:** 2017-02-27

**Authors:** Nelamakanahalli K Suma, Kukkalli K Shashibhushan, VV Subba Reddy

**Affiliations:** 1 Reader, Department of Pediatrics and Preventive Dentistry, V S Dental College & Hospital, Bengaluru, Karnataka, India; 2 Professor, Department of Pediatrics and Preventive Dentistry, College of Dental Sciences, Davangere, Karnataka, India; 3 Director, Department of Pediatrics and Preventive Dentistry, College of Dental Sciences, Davangere, Karnataka, India

**Keywords:** Cavity disinfectant, Chlorhexidine gluconate, Consepsis, Iodine-potassium iodide, Ora5, Shear bond strength.

## Abstract

**Objective:**

Cavity preparation is a surgical procedure that attempts to remove all infected dentin.^1^ Bacteria left beneath the filling material is greatest threat to the pulp. To reduce the potential for residual caries development and sensitivity, an antibacterial solution with the ability to disinfect the prepared tooth surface would be of help.^2^ So this study was conducted to evaluate and compare the effect of dentin disinfection with 2% chlorhexidine gluconate (Consepsis) and 0.3% iodine (Ora5) on shear bond strength (SBS) of self-etch adhesives to dentin.

**Materials and methods:**

Buccal surfaces of 36 caries-free permanent third molars were ground to expose dentin. All specimens were mounted on acrylic block, divided randomly into three groups, namely group I (control), group II (Con-sepsis), and group III (Ora5). After the application of cavity disinfectant and bonding procedures as per manufacturer’s instructions, composite cylinders were built. Then SBS was measured using universal testing machine.

**Results:**

Statistical analysis of the measurements were made using one-way analysis of variance (ANOVA), which showed that when cavity disinfectants (Consepsis and Ora5) were used there was significant reduction in SBS of composite to dentin when compared with that of control group.

**Interpretation and conclusion:**

The results indicate that the use of commercially available cavity disinfectants, Consepsis containing 2% chlorhexidine gluconate and Ora5 containing 0.3% iodine and 0.15% potassium iodide with self-etch adhesive (Adper Prompt), would significantly lower SBS of composite to dentin.

**How to cite this article:**

Suma NK, Shashibhushan KK, Reddy VVS. Effect of Dentin Disinfection with 2% Chlorhexidine Gluconate and 0.3% Iodine on Dentin Bond Strength: An *in vitro* Study. Int J Clin Pediatr Dent 2017;10(3):223-228.

## INTRODUCTION

Cavity preparation is a surgical procedure that attempts to remove all infected dentin prior to placing a restorative material.^1^ Bacteria left beneath the filling material is the greatest threat to the pulp. Bacterial activity may result in increased pulp sensitivity, pulpal inflammation, and secondary caries.^2^ One of the commonest problem across all restorative material is microleakage.^3^ Microleakage has been demonstrated as a factor in hypersensitivity and secondary caries.^4^ To date, no restorative material has been consistently shown to seal and adhere to dentin. The problems associated with microleakage can be magnified by incomplete sterilization of the prepared tooth. In an effort to remove bacteria laden dentin, various dyes have been tested.^5^ A solution of 0.5% basic fuchsin in propylene glycol was used as a caries disclosing dye.^6^ Anderson et al indicated that the cariously affected dentin contained 1,300 times more colony forming units per milligram (CFU/mg) than the dentin that did not take up the dye. However, the dentin containing <10,000 CFU/mg was not disclosed by the dye.

Adhesion to dentin is still under investigation. New-generation dentin adhesives have increased bond strength between composite resins and tooth, thereby resulting in decreased marginal leakage. Decreased marginal leakage avoids bacterial contamination, which in turn decreases the incidence of secondary caries. Secondary caries may also be result of action of bacteria left under restorations.^7^ Thus, after removal of carious dentin, it is important to eliminate any remaining bacteria that may be present on the prepared tooth surface, in the smear layer, at the enamel-dentin junction or in the dentinal tubules.^8^ To reduce the potential for residual caries development and sensitivity, an antibacterial solution with the ability to disinfect the prepared tooth surface would be of great help.^2^ Today, application of disinfectants like chlorhexidine, hypochlorite, and fluoride after tooth preparation and before restoration is gaining wider acceptance in order to eliminate the potential risk of secondary caries.^9^

A potential problem in use of a disinfectant with dentin bonding agents is the possibility of an adverse effect on the bond strength of composite resins.^10^ Thus, the purpose of this *in vitro* study is to determine the effect of two commercially available disinfectants (Consepsis and Ora5) on SBS of composite to dentin.

## MATERIALS AND METHODS

A total of 36 extracted caries-free third molars were collected from the Department of Oral Surgery, College of Dental Sciences, Davangere, Karnataka, India. The samples were cleaned and scaled using ultrasonic scaling unit, the roots were sectioned, and the crowns were stored in saline until further use. The samples were randomly divided into three groups, namely: Group I (control group), group II (Consepsis group), group III (Ora5 group).

**Fig. 1:**
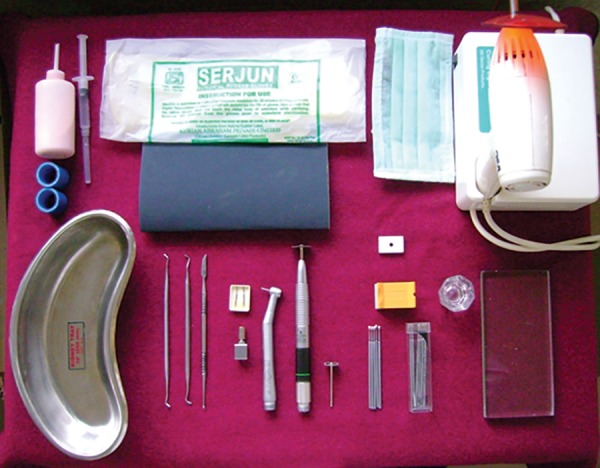
Armamentarium used in the study

Buccal surfaces of all 36 samples were ground flat using diamond cylinder bur with water coolant, until the exposed dentin surface provided 4 mm circular area as bonding site. Then dentin surfaces were polished with 600 grit sand paper in order to obtain smooth surface. All specimens were mounted on acrylic block of 1 inch diameter (Fig. 1).

### Bonding Protocol for Each Group

Group I (control group): Self-etch adhesive (Adper Prompt, 3M ESPE AG Dental Products, Seefeld, Germany) was applied on dentin surface using brush, massage for 15 seconds, applying pressure. Dry thoroughly using gentle stream of air to obtain thin adhesive film. Apply second coat of adhesive using brush, and again dry thoroughly using gentle stream of air. Cure adhesive for 10 seconds.

Group II (Consepsis group): Consepsis cavity disinfectant (lightly flavored 2.0% chlorhexidine gluconate, Ultradent Products, Inc., South Jordan, Utah, USA) was applied on exposed dentin surface using applicator tips provided by the manufacturer, followed by gentle rubbing for 60 seconds, followed by gentle air drying. Self-etch adhesive was then applied as per the manufacturer’s instructions.

**Fig. 2:**
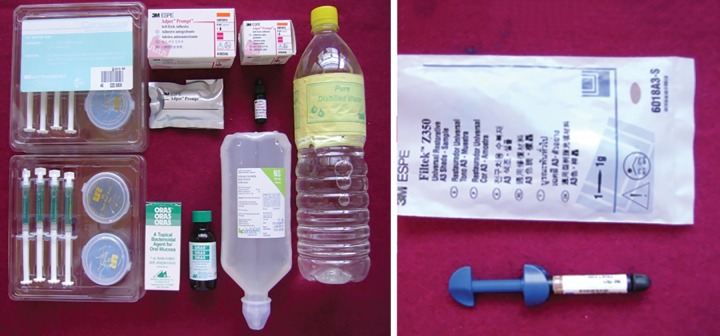
Materials used

Group III (Ora5 group): Ora5 (copper sulfate, iodine, potassium iodide, alcohol 1.5%, McHenry Laboratories, Inc., Texas, USA) was applied using applicator tips on exposed dentin surface for 60 seconds, followed by gentle air drying. Self-etch adhesive was applied over dentin surface as per manufacturer’s instruction (Fig. 2).

**Fig. 3:**
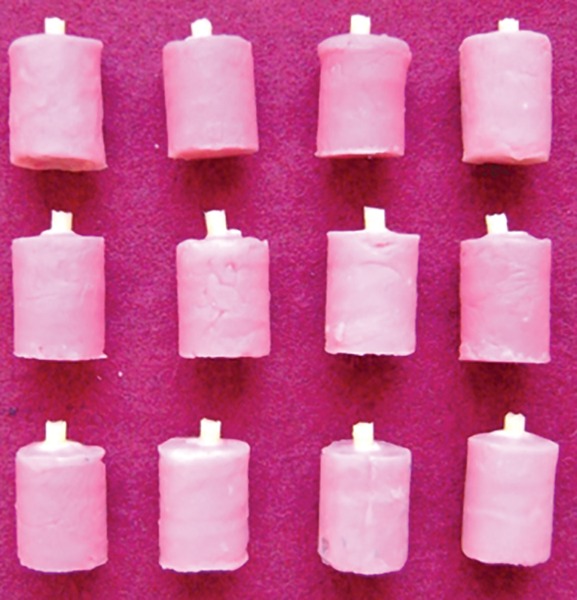
Samples after composite buildup

**Fig. 4:**
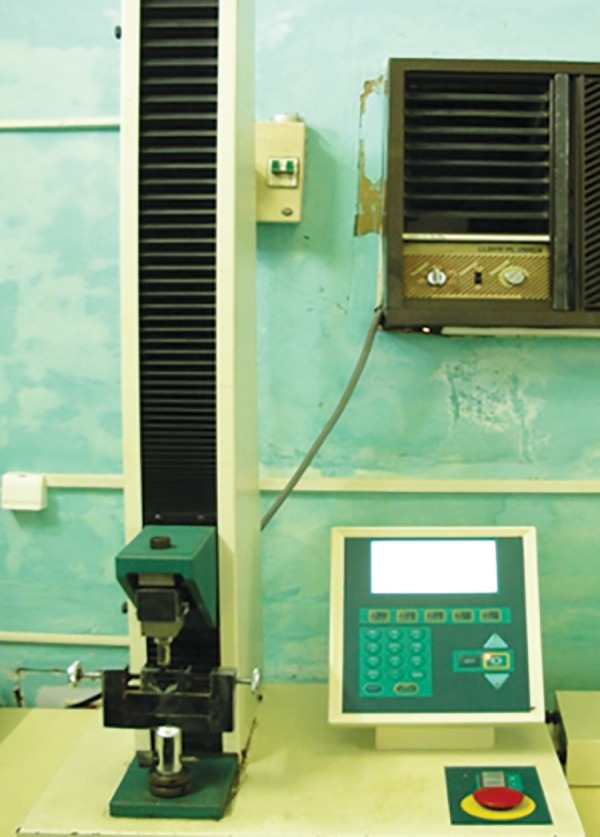
Universal testing machine

For groups I, II, and III, composite (Filtek Z350, 3M ESPE Dental Products, St. Paul, MN, USA) cylinders of 4 mm diameter and 3 mm in height were built incrementally over the bonded dentin surface using a Teflon mold (Fig. 3). All the test specimens were stored in distilled water for 24 hours at 37°C. Then the specimens were subjected for bond strength analysis on Instron testing machine (Fig. 4).

*Shear Bond Strength Analysis:* The specimens were placed in the Instron universal testing machine such that the blade of the machine lied perpendicular to the composite cylinders along the long axis of the crowns.

Force was then applied over the composite cylinders at a crosshead speed of 1 mm/minute unless the cylinders got detached from the dentin surface. The amount of weight needed to detach the composite cylinders was noted and the bond strength was calculated using the formula:

Bond strength = Force in kg needed to debond the composite cylinder × 9.8/total surface area.

Analysis of variance followed by *post hoc* test was used for group-wise comparison of SBS.

## RESULTS

Shear bond strength values (MPa) were calculated from peak load at failure, divided by the surface area of the specimen.

Results are expressed as mean ± standard deviation (SD). One-way ANOVA was used for multiple group comparison followed by *post hoc* test for group-wise comparisons. For all the tests, a p-value <0.001 was used for statistical significance.

Table 1 shows the comparisons of the three groups regarding their range, mean, and median values of SBS in MPa.

**Graph 1:**
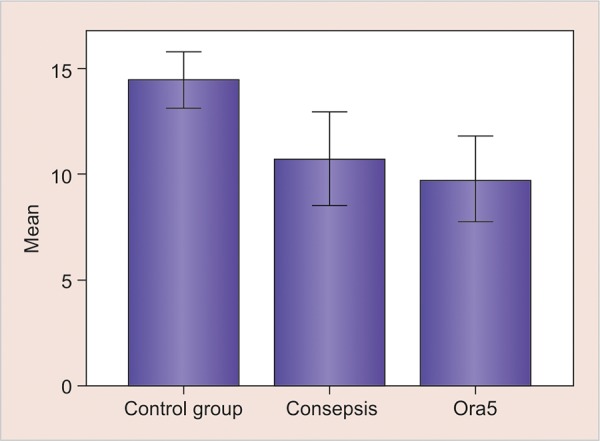
Mean SBS and the SD of test groups

Table 2 and Graph 1 show exclusively the mean SBS and their SD among the test groups. The mean ± SD of group I is 14.46 ± 1.31, of group II is 10.72 ± 2.20, and of group III is 9.76 ± 2.02.

**Table 1:** **Table 1:** Comparison of three groups regarding range, mean, and median values of SBS in MPa

*Study groups (n = 10)*		*Range*		*Mean*		*Median*		*SD*	
Control		11.71-16.34		14.466		14.425		1.310	
Consepsis		7.46-14.34		10.723		10.08		2.205	
Ora5		5.83-12.8		9.765		10.32		2.024	

**Table 2:** **Table 2:** Mean SBS and the SD among test groups

*Study groups*		*Mean± SD*		*Mean difference from control*		F* value		*Significance*		Significant pairs**	
Control		14.46 ± 1.31		–		20.81		p < 0.001, highly significant		1 and II, 1 and III	
Consepsis		10.72 ± 2.20		3.743							
Ora5		9.76 ± 2.02		4.701							

*One-way ANOVA; **Tukey’s HSD test

### Intergroup Comparison

Table 2 shows mean difference and significance by inter-comparing the various groups.

When groups I and II were intercompared, their mean difference was 3.743 MPa with f-value of 20.81 and p-value <0.001, indicating that there was highly significant difference between these two groups.

When groups I and III were intercompared, their mean difference was 4.701 MPa with f-value of 20.81 and p-value <0.001, indicating that there was highly significant difference between these two groups.

Thus, significant pairs were groups I and II, groups I and III when compared using one-way ANOVA and Tukey’s honest significance difference (HSD) test.

At the end of treatment when group I (control group) was compared with group II (Consepsis group), reduction in SBS was highly significant with p < 0.001.

When group I (control group) was compared with group III (Ora5 group) reduction in SBS was highly significant with p < 0.001.

## DISCUSSION

Evaluation of SBS is important as the restoration is subjected to shear stress during mastication. Cavity prepared for restoration is never completely free from microorganisms/sterile, no matter whichever method of caries removal is followed, always a few microorganisms are left behind. Some authors say that once cavity is sealed by restoration, the microorganisms die out. But studies have shown that even for a period of 1 year the microorganisms which are left behind in cavity may be viable and are capable of causing secondary caries in presence of microleakage, hence leading to failure of treatment. Sterilization of prepared cavity is one of Black’s instructions. He has advocated surgical sterilization of dentinal walls before insertion of restorative material. Branstrom and Nyborg were the first to propose the concept of disinfecting the teeth; the recommended agent was benzalkonium chloride-based disinfectant. So some agents that have antimicrobial property have been tried for complete sterilization of prepared cavity. So an *in vitro* study was conducted to evaluate the effect of two commercially available dentin disinfectants on SBS of dentin, namely Consepsis and Ora5. Consepsis is 2% chlorhexidine gluconate (Ultra dent Products, Inc.). Consepsis liquid is indicated before crown cementation, for restorative preparations of crowns, inlays, and composite, and also for procedural endodontic disinfection. Ora5 is topical bactericidal agent, composed of 0.3% iodine, 0.15% potassium iodide, 5.5% copper sulfate, and 1.5% alcohol manufactured by McHenry Laboratories, Inc.

The current generation of disinfectants contains 2% chlorhexidine gluconate as primary active ingredient, in addition to benzalkonium chloride. Chlorhexidine glu-conate is an antiseptic with a wide spectrum of action.^11^ Chlorhexidine is most potent antimicrobial agent to combat *Streptococcus mutans.* It has been found to be effective in reducing the levels of *S. mutans* found in occlusal caries and on exposed root surfaces.^12^

Ora5 is a commercially available iodine-potassium iodide (I_2_-KI)-based oral disinfectant. Several human studies have shown that I_2_-KI solution can reduce *Streptococcus mutans* levels on smooth surface for prolonged intervals. Meiers and Schachtele have investigated the ability of Ora5 to penetrate and kill the bacteria in fissures known to contain incipient caries lesions and reduced the *S. mutans* found in fissures.^13^

In this present *in vitro* study, when cavity disinfectants like Consepsis and Ora5 were used for cavity disinfection (as per manufacturer’s instructions) before application of self-etch dentin bonding agent (Adper Prompt), there was a significant reduction in SBS to dentin, when compared with that of control group. Ora5 group showed greater reduction of SBS than Consepsis group. Similar observation was made by Coa et al,^12^ who found that the disinfectant decreases SBS to dentin. Gurgan et al^14^ showed that using a cavity disinfectant, 2% chlorhexidine before or after acid etching, without rinsing it off, decreases the SBS to dentin; this could be due to cavity disinfectants applied on dentin surfaces that were resistant to acidic conditioning. This acid-resistant layer might inhibit the ability of the hydrophilic resin to impregnate the dentin surface.

Meiers and Kresin^15^ found that the use of cavity disinfection after tooth preparation and before the application of dentin bonding agent could help to reduce the potential of residual caries. They evaluated the effect of two dentin disinfectants, one chlorhexidine-based and the other an iodine/potassium copper sulfate solution (Ora5) and found that both Ora5 and chlorhexidine gluconate adversely affected SBS of composite to dentin mediated by Syntac, but did not affect that mediated by Tenure. They also concluded that the effect of cavity disinfectants on SBS of composite to dentin treated with dentin bonding resin was material-specific regarding their interactions with various dentin bonding system ability to seal dentin. However, the combination of Ora5 with Syntac did significantly increase gingival microleakage levels. This may be indicative of some negative interaction between the iodide/potassium iodide, copper sulfate solution, and the primer or adhesive of Syntac. This would lead to speculation that the chemical residue left from Ora5 may have contributed to decrease in wettability of the adhesive and a resultant decrease in its ability to impregnate the dentin surface. Scanning electron microscopy examination of chlorhexidine-treated smear layers was less affected by the dentin primer of Syntac and conditioner of Tenure, indicating this treated smear layer was made acid-resistant. However, the iodine/potassium iodide, copper sulfate-treated smear layers did not show the same resistance to removal or modification as did by the chlorhexidine.

Tulunoglu et al^10^ in an *in vivo* study found that chlorhexidine cavity disinfectant increases microleak-age scores when used prior to the implementation of Syntac and prime and bond dentin adhesive systems. They stated that there might have been some negative interaction between the cavity disinfectants and dentin bonding agents.^11^

Ricardo concluded that there was significantly lower SBS when 2% chlorhexidine solution (Cav Clean) was used, which was suggested may be due to the fact that remnants of chlorhexidine could interact with calcium and phosphate present in dentin and therefore, inhibit the bonding ability.^11^ da Silva Telles reported that most of the restorations (resin composite and compomer) bonded with Prompt L-Pop exhibited interfacial gaps. However, no interfacial gap formation was observed in the resin composite restoration bonded with Prompt L-Pop and Clearfil SE Bond, in his study. Only specimens treated with Ora5 exhibited gap formation at the tooth and resin composite interface, regardless of dentin bonding resins used. Moreover, in these specimens, no resin tag formation was observed. This may depend on the fact that chemical residue left from Ora5 has contributed to a decrease in wettability of dentin bonding resins and a resultant decrease in its ability to impregnate to the dentin surface.^6^

Recently, Pilo et al^16^ indicated that Consepsis when applied after etching and washed off could increase the SBS of One Step. Washing off the chlorhexidine that contains a surfactant might only partially drive away the chlorhexidine molecules and the bound molecules can serve as a cosurfactant on the conditioned dentin before resin is applied.^17^ Murat and Ferit^18^ suggested that cavity disinfectants can improve the sealing ability of dentin bonding agents by remoistening the cavity prior to placing a dentin bonding agent that bonds to damp tooth structure. Schaeken et al^19^ have claimed that bound chlorhexidine molecules might serve as a co-surfactant on dentin surface. Benzalkonium chloride has also shown to link the collagen and does not impair hybridization.^2^

Perdigao and others used the ALL Bond 2 adhesive system (BISCO) and found that pretreatment with chlorhexidine had no significant effect on SBS of composite to dentin. However, as the chlorhexidine was not washed off the dentin, debris remaining on the dentin surface and in the tubules may account for decrease in strength. Meiers and Kresin^15^ found that cavity washing with 2% chlorhexidine did not affect SBS/microleakage of composite resins, since chlorhexidine was applied before etching; its effect on bond strength values could have been neutralized by etching process.^17^

The results of the present study indicate that the use of commercially available cavity disinfectants, Consepsis containing 2% chlorhexidine gluconate and Ora5 containing 0.3% iodine, 0.15% potassium iodide with self-etch adhesive (Adper Prompt) would significantly lower the SBS of composite to dentin.

So, further long-term clinical studies are required to evaluate the efficacy and effect of cavity disinfectants on dentin bond strength.
